# Panniculectomy for a Rare End-Stage Complication of Severe Obesity and Increasing Surgical Phenomenon

**DOI:** 10.7759/cureus.57592

**Published:** 2024-04-04

**Authors:** Nelson Chen, Tessa K Daly, Wei Ming Ooi

**Affiliations:** 1 General Surgery, Northern Hospital Epping, Melbourne, AUS

**Keywords:** panniculectomy, abdominal pannus, lymphoedema, obesity, panniculus morbidus

## Abstract

Panniculus morbidus (PM) is a presentation of severe chronic abdominal lymphoedema associated with obesity resulting in oedema and chronic fibrosis. It is a multifaceted condition with significant clinical and psychosocial implications.

A 29-year-old female weighing 260 kg with a body mass index of 95 kg/m^2^ had recurrent infections and sepsis associated with an abdominal pannus extending to her knees and an area of ulceration. The pannus was indurated with extensive fibrosis that significantly affected her quality of life (QOL) requiring assistance for all activities of daily living (ADLs). A panniculectomy was performed with a negative pressure skin dressing over the skin wound. She was discharged after two days. Two months postoperatively, she reported significant improvement in QOL and can now mobilise and perform ADLs independently with no recurrent admissions.

The global prevalence of obesity is reaching pandemic proportions and so will its complications. It can be functionally debilitating and worsen obesity. Surgical resection is indicated to restore mobility and function, prevent recurrent infections, improve QOL, and reduce economic burden. Patients report high satisfaction rates following surgery. Panniculectomy is an effective treatment to alleviate morbidity in severe obesity and should be considered in patients with recurrent infections and a significant impact on QOL.

## Introduction

Panniculus morbidus (PM) is a rare presentation of severe chronic abdominal lymphoedema associated with obesity [1]. Excessive adipose tissue accumulation creates a pendulous overhanging fold of fat and skin resulting in lymphatic obstruction and irreversible fibrosis [1]. PM is a multifaceted condition with significant clinical and psychosocial implications resulting in a sedentary lifestyle and progressive health deterioration which contributes to further difficulty achieving weight loss.

The global prevalence of obesity has tripled between 1975 and 2016 to reach pandemic proportions, with the prevalence of overweight and obesity affecting 60% of adult Australians [2,3]. Although obesity has historically been considered a Western issue, rates are on the rise in every country due to economic growth, ‘westernisation’ of dietary habits, and urbanisation [4]. As obesity rates rise, such presentations will become increasingly common.

We present the case of a 29-year-old female with PM with a non-healing ulcer on the underside causing recurrent sepsis and significant psychosocial complications managed successfully with panniculectomy to illustrate the role of surgery in this cohort of patients.

## Case presentation

**Figure 1:**
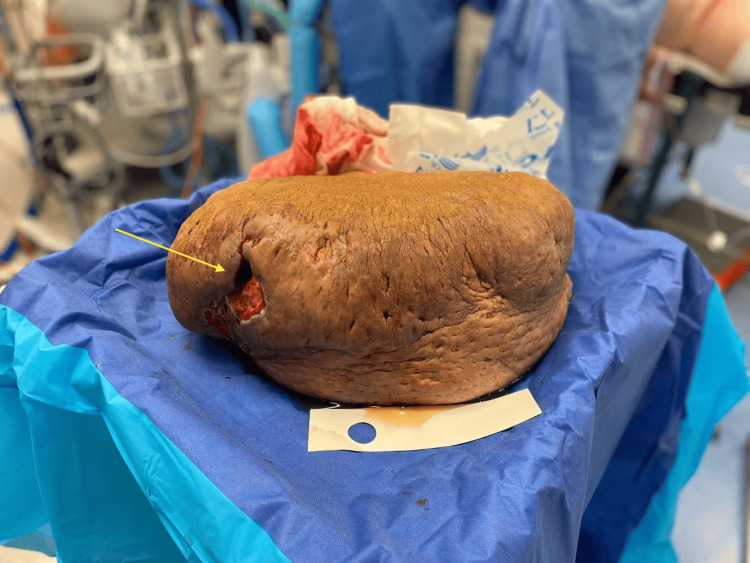
Photograph demonstrating the pannus measuring 43 x 23 x 18 cm with a large ulcer on the dependent portion (arrow) and significant cobblestone peau d’orange appearance of the skin.

A non-diabetic 29-year-old female with a body mass index of 95 kg/m^2^ who took no regular medications was referred for recurrent infections of her abdominal pannus extending to her knees that was enlarging with an associated non-healing necrotic ulcer. She was febrile, tachycardic, and hypotensive with an elevated white cell count and C-reactive protein of 412 mg/L on this presentation. She weighed 260 kg and had a height of 165 cm. Examination revealed a large, firm, and indurated pannus with a peau-d’orange appearance and extensive fibrotic skin changes. There was a large ulcer with necrotic skin along the dependent portion secondary to pressure necrosis from the weight of the pannus itself (Figure 1).

The pannus had been reportedly progressively enlarging over four years causing increasingly significant morbidity to the patient and her family, limiting her mobility, personal hygiene, and social life. She required assistance with all activities of daily living (ADLs). She improved with vancomycin and tazocin and returned several weeks later with another infection.

A panniculectomy was offered due to the non-healing chronic ulcer with recurrent infections and the significant functional and psychosocial effects of the pannus. The patient was positioned supine on a bariatric operating table with adequate pressure supports. An elliptical incision was drawn to create a flat, tension-free closure of the skin flaps. The pannus was stretched superiorly to mark the inferior limits of the incision and then inferiorly to mark the superior limits taking care to avoid bony landmarks and ensuring healthy unaffected skin at the base of the pannus. Retraction and elevation of the pannus were facilitated by three assistants due to the significant weight of the pannus.

Dissection through the subcutaneous tissues to the base of the pannus was performed with a combination of cautery and a Ligasure™ device. Multiple large superficial veins up to 15 mm required suture ligation. The pannus was dissected down to fascia without undermining the incision to avoid devascularisation of the skin flaps. A large suction drain was placed at the wound bed to reduce the risk of seroma formation. The wound was closed in multiple layers to reduce dead space. The skin was approximated with a combination of staples and vertical mattress sutures. A negative pressure skin dressing was placed over the wound. Operative time was 120 minutes. The specimen was unfortunately not weighed due to its size but the patient was approximately 10 kg lighter on postoperative day one.

The patient was discharged postoperative day two with minimal pain and was followed up in the clinic for removal of the negative pressure skin dressing at two weeks. The staples and sutures were removed at four weeks with a minor dehiscence at the midline that healed with time. Since the panniculectomy, the patient reports significant improvement in quality of life (QOL). She is now independently mobile without assistance, can attend to her ADLs, and reported an increase in social activity with no further admissions related to the pannus.

**Figure 2:**
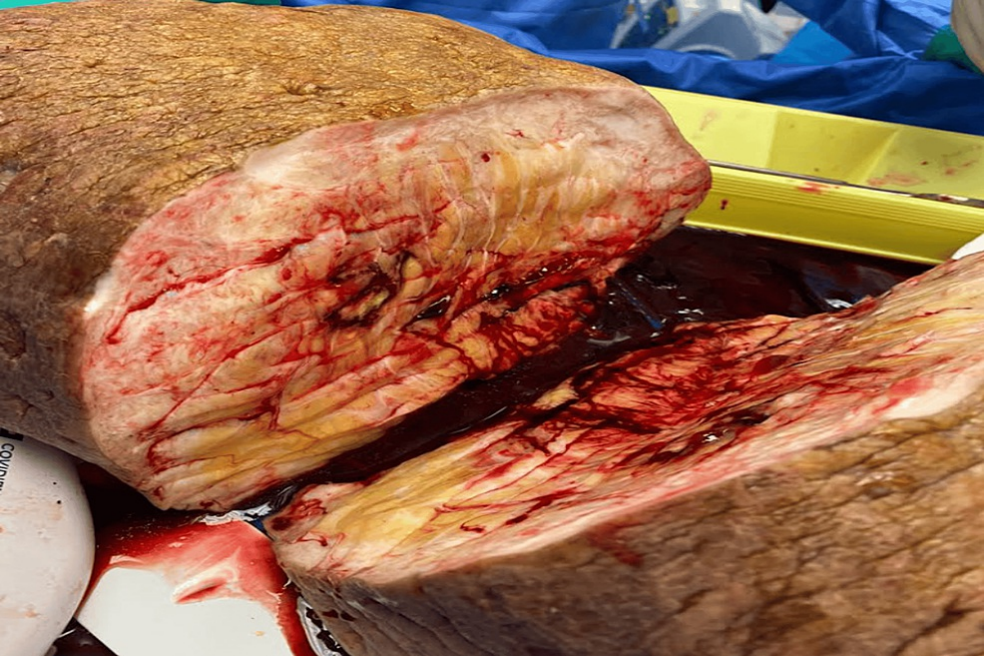
Photograph of the specimen cut in half to demonstrate extensive chronic dermal fibrosis of the subcutaneous soft tissue.

Histological examination found extensive dermal fibrosis and fat necrosis (Figure 2). There was a widening of the fibrous septa, with numerous reactive fibroblasts and histiocytes throughout. Adjacent to this was nested squamous epithelium with associated foreign body giant cell response.

## Discussion

PM was first described as a distinct clinical and pathological entity by Farshid and Weiss in 1998 [1], characterised by a large overhanging abdominal pannus complicated by significant lymphoedema. It is also known as abdominal elephantiasis, massive localised lymphoedema, and monstrous tumefactive pseudosarcoma [5-7]. The gravitational pull of the pendulous overgrowth of benign adipose tissue results in obstruction of the lymphatic and valveless superficial venous system resulting in oedema [6,8]. Accumulation of protein-rich interstitial fluid causes a local inflammatory response with increased macrophage activity and fibroblast migration resulting in collagen deposition and fibrosis and eventually a cobblestone or peau d’orange appearance of the dermis [8].

PM can have a significant impact on a patient’s physical, psychological and social well-being [5,8]. It can reduce mobility and independent function and its visible appearance may also contribute to social isolation and a sedentary lifestyle, resulting in patients being housebound or bedbound and worsening obesity [5,8,9]. It also impairs personal hygiene and toileting and reduces self-esteem which may lead to depression [5].

Surgical resection is indicated to restore mobility and function, prevent recurrent infections, and improve QOL [6,7]. It also reduces economic burden by reducing the ongoing requirements for nursing and wound care, social care expenses, and admissions for infections and other complications [10]. Elevation of the pannus perpendicularly is an important consideration of the procedure for the safety of the patient and staff [11,12]. It also facilitates operative access and helps drain the pannus’s lymphatic and venous blood to decrease blood loss [10]. Multiple techniques have been described, including the use of towel clips, Rush nails or Steinman pins paired with a traction bar, and a pulley system or hydraulic lift to elevate the pannus preoperatively [11]. Manual elevation with multiple assistants can be difficult but can be considered an option in the absence of other equipment. The elevation is important because the heaviest pannus to be reported in the literature weighed 94 kg [11]. Large operations such as this result in large volumes of dead space, so it is also important to avoid resecting too much fat and undermining the abdominal wall [12]. Closure should be performed in layers such as in ours to reduce the incidence of seroma and wound breakdown, along with the consideration of a negative pressure wound dressing [12].

Surgeons should be mindful that the patient population is inherently a high-risk cohort for surgery, with complication rates ranging from 15% to 80% [5,10]. Wound-related complications are most common, including seroma, dehiscence, fat necrosis, infection, and haematoma [5,10]. The learning curve for massive panniculectomy is steep and complication rates may be higher at the outset in this uncommon operation in a high-risk population [12]. Felmerer et al. and Koulaxouzidis et al. suggested that preoperative complex decongestive physiotherapy (CDP) is associated with reduced complication rates compared to the control group without CDP [9,13]. Despite this, due to the large psychosocial and functional consequence of this pathological entity, patients still report high satisfaction rates following surgical intervention such as in our patient [5].

## Conclusions

PM is a consequence of severe chronic abdominal lymphoedema associated with morbid obesity. Its prevalence is expected to rise alongside obesity in the future. This case illustrates that surgical treatment with panniculectomy can be a safe and effective treatment to alleviate the morbidity and mortality associated with this condition and improve the physical, psychological, and social well-being of patients.
